# Case Report: Concurrent autoimmune gastritis and high-grade dysplasia in a gastric hyperplastic polyp

**DOI:** 10.3389/fmed.2025.1716969

**Published:** 2026-01-12

**Authors:** Tianwa Wang, Jinfeng Luo, Yajing Han, Qiu Yang, Shusong Peng

**Affiliations:** 1 Department of Pathology (Longhua Branch), Shenzhen People’s Hospital (The Second Clinical Medical College, Jinan University), Shenzhen, Guangdong, China; 2 Department of Gastroenterology, Peking University Third Hospital, Beijing, China; 3 Department of Gastroenterology (Longhua Branch), Shenzhen People’s Hospital (The Second Clinical Medical College, Jinan University), Shenzhen, Guangdong, China

**Keywords:** autoimmune gastritis, corpus-predominant gastritis, gastric hyperplastic polyp, high-grade dysplasia (hgd), hypergastrinemia

## Abstract

Autoimmune gastritis (AIG) is an under-recognized, corpus-predominant autoimmune loss of oxyntic glands that creates a metaplastic, inflamed field in which hyperplastic polyps are common, while epithelial dysplasia is uncommon but management-defining. We report a 35-year-old woman with corpus-predominant atrophy and three pedunculated polyps removed by endoscopic mucosal resection (EMR); initial pathology called a hyperplastic polyp with focal low-grade dysplasia (LGD). On tertiary review, the background was identified as AIG (corpus-restricted oxyntic atrophy with pseudopyloric, also called oxyntic, metaplasia and mild intestinal metaplasia) and a small polyp-head focus was upgraded to high-grade dysplasia (HGD); margins were negative. *Helicobacter pylori* was negative on histology/immunohistochemistry and urea breath testing. Targeted serology at index and follow-up showed persistently elevated anti-parietal-cell antibodies and fasting gastrin, with intrinsic-factor antibody negative and vitamin B_12_ within range. Early follow-up confirmed a healed EMR site with a persistent corpus-predominant background, and a small body polyp that proved to be a hyperplastic polyp without dysplasia. This case highlights that concurrent AIG and HGD shift management from routine post-polypectomy care to definitive excision plus corpus-focused surveillance, and it argues for actively considering AIG when dysplasia is found in a body polyp—and considering dysplasia when AIG is present.

## Introduction

Autoimmune gastritis (AIG) is an under-recognized, corpus-predominant autoimmune inflammation of the gastric mucosa that leads to progressive loss of oxyntic glands, hypochlorhydria, and secondary hypergastrinemia (1–3). Although AIG is often under-recognized, it has been linked to various gastric lesions, particularly hyperplastic polyps (4–6).

We report a 35-year-old woman with autoimmune gastritis (AIG) and a hyperplastic polyp, in whom expert pathology identified high-grade dysplasia (HGD) in the polyp following endoscopic mucosal resection (EMR). This case underscores the need for clinicians and pathologists to actively consider AIG when dysplasia is found in gastric polyps and to carefully evaluate for dysplasia in patients with AIG.

## Case description

A 35-year-old woman presented with upper-abdominal bloating, dyspepsia, and poor appetite. She reported no long-term medications or proton-pump inhibitor use, and no personal or family history of autoimmune disease. Routine checkups confirmed normal thyroid function, with TSH, FT4 and FT3 within reference ranges, as summarized in Table 1 (7–9).

**TABLE 1:** Serologic and ^13^C-urea breath test results at initial presentation and follow-up.

Test	Method	Reference range	Initial (Oct 2024)	Follow-up (May 2025)
Gastrin-17, pmol/L	CLIA^1^	1.50–7.50	**68.91**	**53.36**
Anti-parietal cell antibody (PCA-IgG), U/mL	CLIA	<20	**350.07**	**320.30**
Anti-intrinsic factor antibody (IFA), U/mL	CLIA	<20	< 0.50	4.14
^13^C-urea breath test (ΔDOB), %	CLIA	<4.0	0.6	NA
Hemoglobin (HGB), g/L	Automated CBC^2^	115–150	**110**	**106**
Vitamin B12, pmol/L	CLIA	141.9–611.2	435	333.19
Total T3, nmol/L	CLIA	1.25–2.35	2.34	1.83
Total T4, nmol/L	CLIA	75.0–150.0	**164.96**	130.76
Thyroid-stimulating hormone (TSH), μIU/mL	CLIA	0.60–4.90	1.06	1.00

^1^CLIA, chemiluminescence immunoassay.

^2^CBC, complete blood count. ΔDOB, delta over baseline; NA, not available. Initial tests were performed at first presentation (October 2024); follow-up tests were performed at repeat evaluation (May 2025). Reference ranges are those of the institutional laboratory. Values in bold are outside the reference range.

**FIGURE 1:**
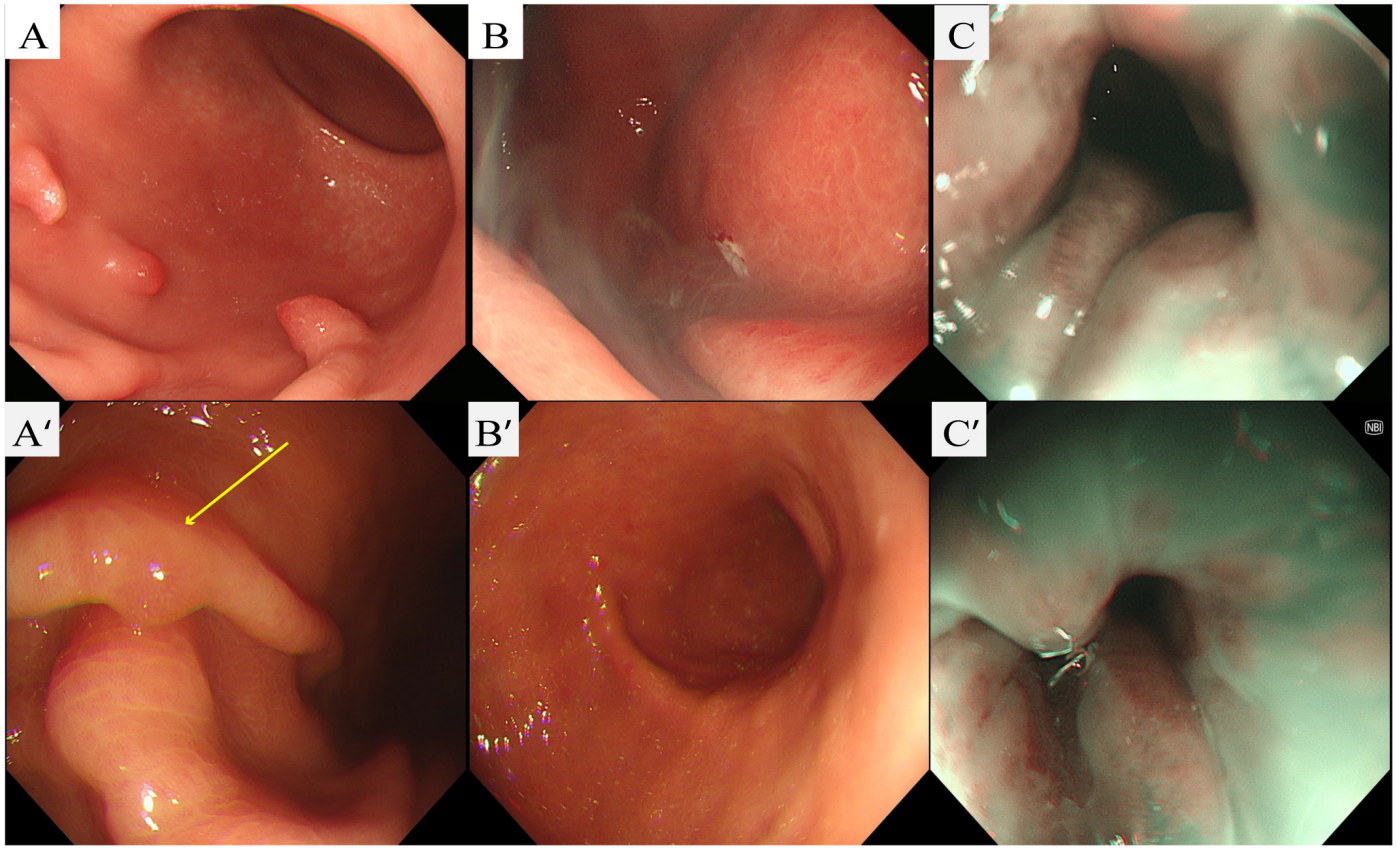
Initial and follow-up upper endoscopy. **(A)** Initial endoscopy (white-light imaging, WLI): corpus-predominant atrophic background with multiple pedunculated polyps; the regular arrangement of collecting venules (RAC) is absent. **(B)** Immediately after endoscopic mucosal resection (EMR) at the initial session: snare electrocautery with a post-resection cautery plume at the polyp stalk. **(C)** Initial narrow-band imaging (NBI): attenuated pit pattern and prominent subepithelial vessels compatible with oxyntic atrophy. **(A’)** Follow-up endoscopy (WLI): yellow arrow marks a small polyp along the mid-body greater curvature. **(B’)** Follow-up (WLI): site of the prior polypectomy showing a well-healed post-EMR scar at the original stalk/base. **(C’)** Follow-up NBI: persistent corpus-predominant atrophic background with altered pit/vascular architecture.

**FIGURE 2:**
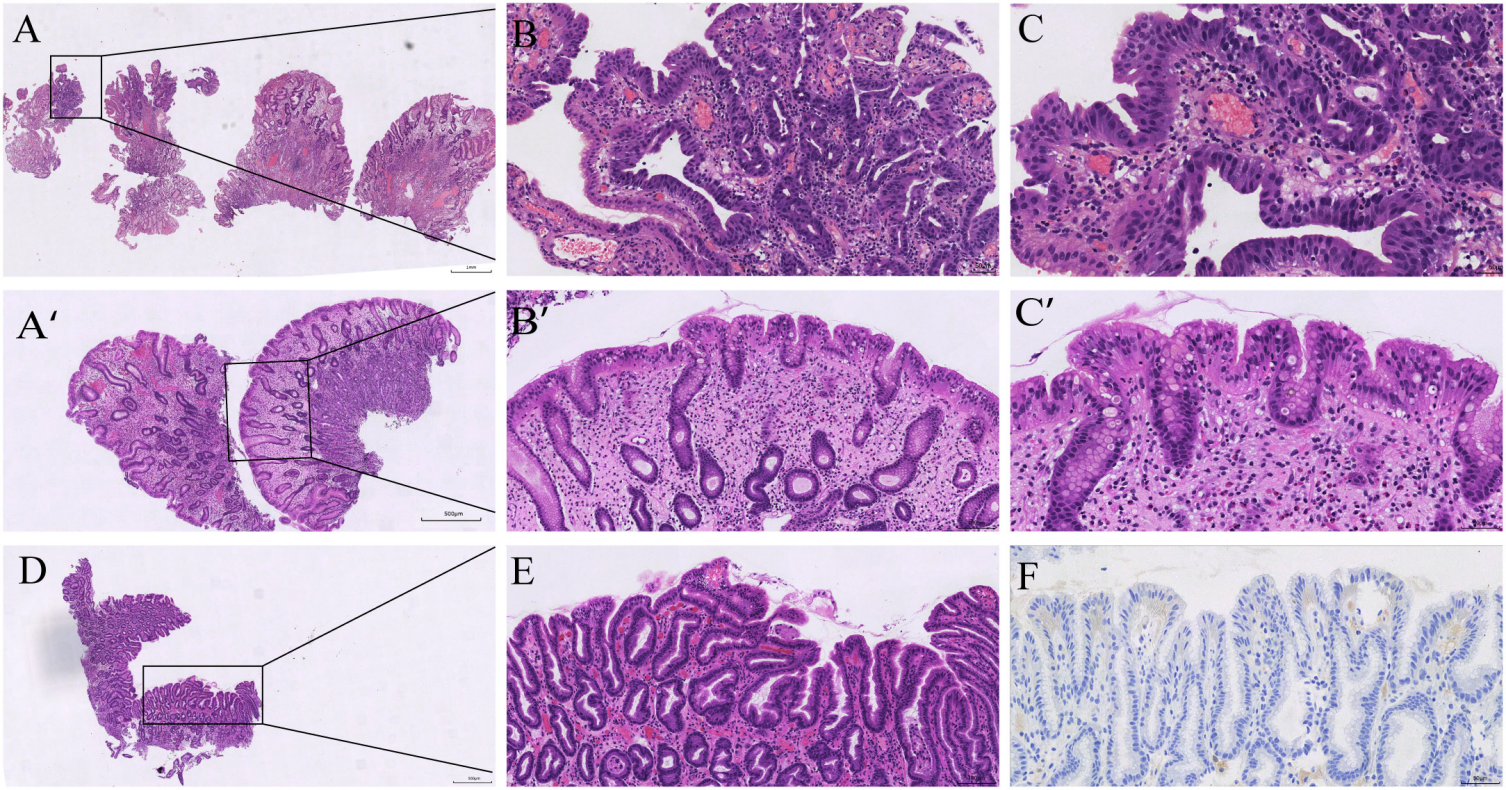
Histopathology from the initial and follow-up procedures. **(A–C)** Initial endoscopic mucosal resection (EMR) specimen from the gastric body: hyperplastic polyp with a focal high-grade intraepithelial neoplasia (HGD) in the polyp head; the background corpus mucosa shows oxyntic atrophy with pseudopyloric/oxyntic metaplasia and mild intestinal metaplasia (H&E, low→high power). **(A’–C’)** Follow-up body polyp: hyperplastic polyp without dysplasia on a background compatible with AIG (H&E, low→high power). **(D–E)** Follow-up antral mapping biopsy: mild chronic gastritis with focal intestinal metaplasia (H&E, low→high power). **(F)** Helicobacter pylori immunohistochemistry negative (antrum).

At the initial upper endoscopy, the gastric body showed diffuse punctate erythema with loss of the regular arrangement of collecting venules; three pedunculated polyps (∼6–10 mm) along the lower-body greater curvature were snare-resected en bloc in the same session (Figures 1A–C), and the entire EMR material was submitted for histologic examination, on which local pathology reported hyperplastic polyp with focal low-grade dysplasia (LGD) and did not assign a specific background gastritis. Because the endoscopic appearance suggested a corpus-predominant process in a young patient and the background was not characterized locally, slides were referred to Peking University Third Hospital for expert review. On consultation, the background body mucosa was diagnosed as autoimmune gastritis (AIG) and the polyp-head focus was upgraded to high-grade dysplasia (HGD; Vienna category C4) with negative margins. The AIG diagnosis was supported by diffuse oxyntic gland loss with parietal-cell depletion and replacement by tightly packed pyloric-type mucous glands (pseudopyloric metaplasia), with patchy intestinal metaplasia and relative antral sparing; *H. pylori* was negative on histology and immunohistochemistry (Figures 2A–C).

At follow-up in May 2025, endoscopy showed a well-healed EMR scar and a persistent corpus-predominant pattern (Figures 1A’–C’). A single ∼5-mm sessile body polyp was removed. Histology again showed a hyperplastic polyp without dysplasia on a body background compatible with AIG (Figures 2A’–C’). Mapping biopsies from the antrum demonstrated mild chronic gastritis without *H. pylori* on immunohistochemistry (Figures 2D–F).

**FIGURE 3:**
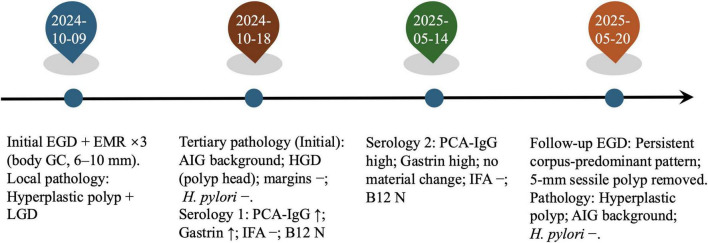
Timeline@@@ of the clinical course. Initial EGD with endoscopic mucosal resection (EMR) of three body polyps (6–10 mm); local pathology: hyperplastic polyp with focal low-grade dysplasia (LGD). Tertiary review of the initial specimen: corpus-restricted autoimmune gastritis (AIG) and high-grade dysplasia (HGD) in the polyp head; margins negative; *H. pylori* negative. Targeted serology at baseline and again at follow-up showed persistently high PCA-IgG and fasting gastrin with IFA negative and vitamin B_12_ within range; mild anemia was present. Follow-up EGD: persistent corpus-predominant pattern; a 5-mm sessile body polyp was removed and read as a hyperplastic polyp on an AIG background; *H. pylori* immunohistochemistry negative.

After histology prompted targeted testing, baseline serology in October 2024 showed gastrin-17 68.91 pmol/L and anti-parietal-cell antibody 350.07 U/mL, while intrinsic-factor antibody was negative and vitamin B_12_ remained within range. Hemoglobin was 110 g/L, indicating mild anemia. The ^13C-urea breath test was negative (Table 1). These findings supported AIG. At follow-up in May 2025, serology showed no material change (persistently above reference), with intrinsic-factor antibody remaining negative and vitamin B_12_ within range. No additional therapy was required beyond corpus-focused surveillance. The overall clinical course, including diagnostic evaluations and longitudinal follow-up, is summarized in Figure 3.

## Discussion

This case highlights the rare coexistence of AIG and HGD in 35-year-old woman, significantly altering management strategies. Although AIG is frequently associated with benign gastric lesions, the presence of HGD in a hyperplastic polyp is an unusual and clinically significant finding (4, 5). This case underlines the importance of considering AIG as a background condition in patients with gastric polyps and emphasizes the need for a more vigilant approach when dysplasia is detected in such patients (1, 2, 10). This case places the spotlight on comorbidity: HGD arising within a gastric hyperplastic polyp in an AIG field. Understanding how and why these two processes intersect explains both the presentation and the management (2).

Autoimmune gastritis’s pathophysiology involves chronic inflammation and oxyntic gland atrophy, leading to a metaplastic gastric environment that predisposes to polyp formation (1–3, 11). These polyps are typically benign; however, sustained inflammation and repeated regeneration can create diffuse mucosal susceptibility, with cumulative genomic stress that increases the risk of dysplasia (6, 12, 13). Our case suggests that the chronic inflammation and low-acid environment in AIG provide a plausible milieu for this progression, as evidenced by HGD developing within a hyperplastic polyp (5, 11).

Although the gastrin axis in AIG is classically linked to enterochromaffin-like (ECL) cell hyperplasia and type I gastric neuroendocrine tumors, our case suggests that AIG can also foster epithelial neoplasia via an inflammation-metaplasia-dysplasia pathway, resulting in HGD (1, 10, 14). The histologic findings in our patient, particularly pseudopyloric metaplasia with intestinal metaplasia, fit this framework (1, 3). This underscores the need for heightened awareness among clinicians and pathologists regarding the potential for dysplasia in AIG patients, even in seemingly benign lesions such as hyperplastic polyps (5, 15).

Compared with prior reports, high-grade dysplasia within a gastric hyperplastic polyp on an AIG background appears rare, which makes this co-morbidity clinically notable (16, 17). Although direct prospective data linking AIG itself to gastric adenocarcinoma are limited, the American Gastroenterological Association (AGA) Clinical Practice Update on Atrophic Gastritis (2021) classifies advanced atrophic gastritis as a preneoplastic condition and recommends surveillance, and a recent clinical review by Castellana et al. (2) synthesizes evidence that autoimmune atrophic gastritis carries increased neoplastic potential and outlines practical management (2, 15, 18). On this backdrop, our case documents epithelial HGD within a hyperplastic polyp in AIG, reinforcing complete excision and corpus-focused follow-up (10, 15).

This case also highlights the importance of early detection and intervention in patients with AIG and gastric polyps. As dysplasia in these patients may be subtle, expert pathology review is crucial for accurate diagnosis (3, 15). Once HGD is excised with clear margins, the management focus should shift from the polyp itself to the underlying background disease. AIG patients, particularly those with dysplastic lesions, should undergo short-interval re-inspections (6–12 months) with thorough gastric corpus inspection, a lower threshold for endoscopic resection of new lesions, and targeted biopsies to monitor background atrophy and metaplasia (10, 15, 18). In addition to short-interval endoscopic surveillance, longer-term follow-up is planned, including annual endoscopy and periodic serologic monitoring (gastrin, parietal cell antibody, intrinsic factor antibody, vitamin B_12_, and hemoglobin), in accordance with guideline-based management of AIG.

Future research should focus on better understanding the molecular mechanisms underlying dysplasia in AIG and developing biomarkers for early detection. Additionally, large-scale studies are needed to establish more detailed guidelines for surveillance in AIG patients with gastric polyps, as well as to identify optimal intervention strategies to prevent malignant progression.

## Conclusion

This case demonstrates that recognizing AIG as the background disease can be as important as grading the lesion itself. In a young adult with a gastric body hyperplastic polyp, expert pathology identified corpus-restricted AIG and HGD at the polyp head—a combination that immediately changed management from routine post-polypectomy care to definitive excision plus corpus-focused surveillance. The practical lesson is simple: when endoscopy shows a corpus-predominant atrophic pattern with polyps, think AIG, look for pseudopyloric (oxyntic) metaplasia and surface pit hyperplasia, and scrutinize the polyp head for loss of maturation signaling HGD. Anchoring the diagnosis in these field-and-focus principles helps clinicians avoid under-recognition of AIG and ensures that surveillance intensity matches the true biological risk.

## Data Availability

The original contributions presented in this study are included in this article/supplementary material, further inquiries can be directed to the corresponding authors.
